# Reference genome of the color polymorphic desert annual plant sandblossoms, *Linanthus parryae*

**DOI:** 10.1093/jhered/esac052

**Published:** 2022-09-15

**Authors:** Ioana G Anghel, Sarah J Jacobs, Merly Escalona, Mohan P A Marimuthu, Colin W Fairbairn, Eric Beraut, Oanh Nguyen, Erin Toffelmier, H Bradley Shaffer, Felipe Zapata

**Affiliations:** Department of Ecology and Evolutionary Biology, University of California, Los Angeles, Los Angeles, CA, United States; Department of Botany, California Academy of Sciences, San Francisco, CA, United States; Department of Biomolecular Engineering, University of California Santa Cruz, Santa Cruz, CA, United States; DNA Technologies and Expression Analysis Core Laboratory, Genome Center, University of California-Davis, Davis, CA, United States; Department of Ecology and Evolutionary Biology, University of California, Santa Cruz, Santa Cruz, CA, United States; Department of Ecology and Evolutionary Biology, University of California, Santa Cruz, Santa Cruz, CA, United States; DNA Technologies and Expression Analysis Core Laboratory, Genome Center, University of California-Davis, Davis, CA, United States; Department of Ecology and Evolutionary Biology, University of California, Los Angeles, Los Angeles, CA, United States; La Kretz Center for California Conservation Science, Institute of the Environment and Sustainability, University of California, Los Angeles, Los Angeles, CA, United States; Department of Ecology and Evolutionary Biology, University of California, Los Angeles, Los Angeles, CA, United States; La Kretz Center for California Conservation Science, Institute of the Environment and Sustainability, University of California, Los Angeles, Los Angeles, CA, United States; Department of Ecology and Evolutionary Biology, University of California, Los Angeles, Los Angeles, CA, United States

**Keywords:** California Conservation Genomics Project, CCGP, desert plant, Polemoniaceae, polymorphism

## Abstract

Sandblossoms, *Linanthus parryae* is a widespread annual plant species found in washes and sandy open habitats across the Mojave Desert and Eastern Sierra Nevada of California. Studies in this species have played a central role in evolutionary biology, serving as the first test cases of the shifting balance theory of evolution, models of isolation by distance, and metrics to describe the genetic structure of natural populations. Despite the importance of *L. parryae* in the development of landscape genetics and phylogeography, there are no genomic resources available for the species. Through the California Conservation Genomics Project, we assembled the first genome in the genus *Linanthus*. Using PacBio HiFi long reads and Hi-C chromatin conformation capture, we assembled 123 scaffolds spanning 1.51 Gb of the 1.96 Gb estimated genome, with a contig N50 of 18.7 Mb and a scaffold N50 of 124.8 Mb. This assembly, with a BUSCO completeness score of 88.7%, will allow us to revisit foundational ideas central to our understanding of how evolutionary forces operate in a geographic landscape. In addition, it will be a new resource to uncover adaptations to arid environments in the fragile desert habitat threatened by urban and solar farm development, climate change, and off-road vehicles.

## Introduction

**Fig. 1.**
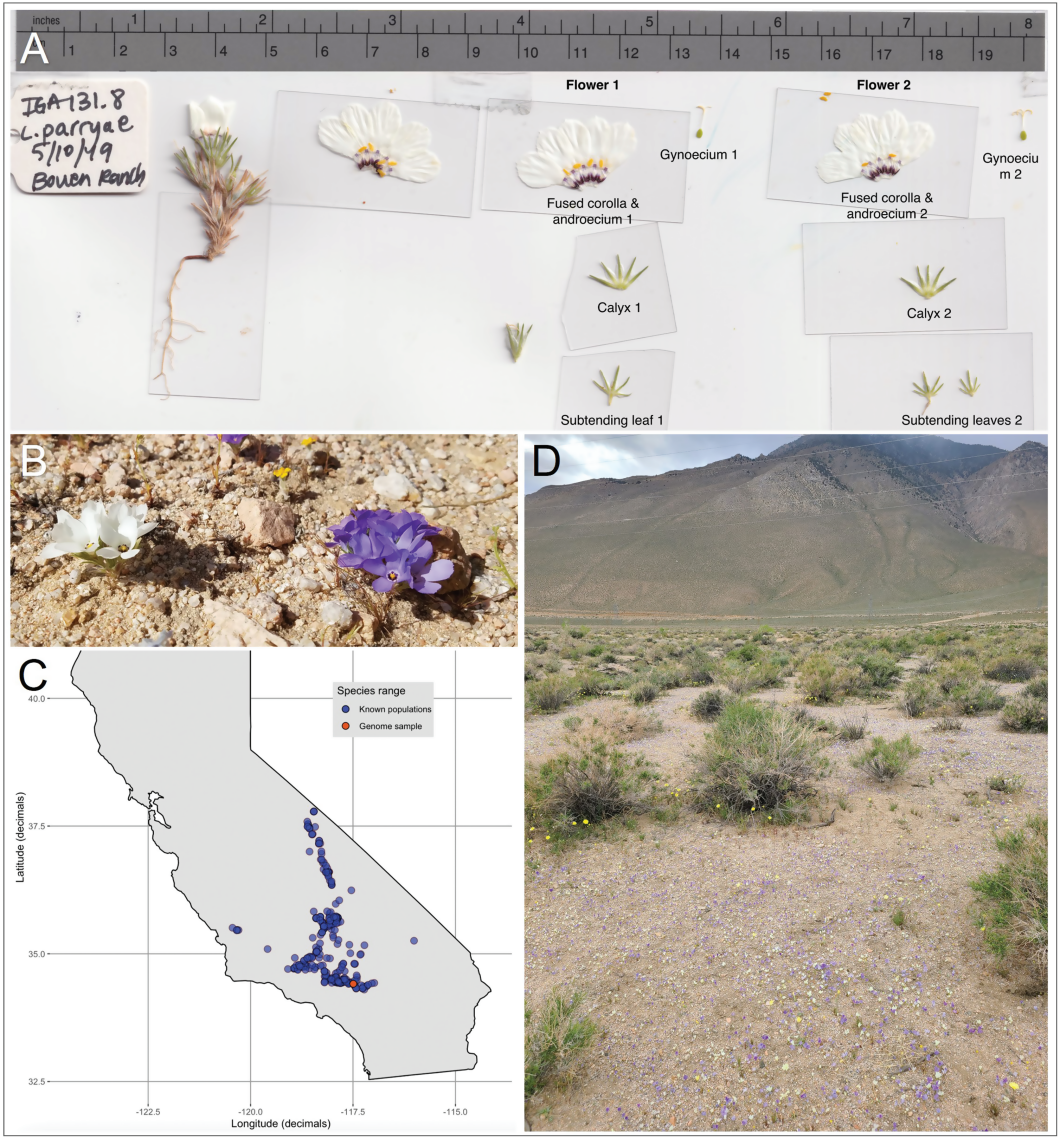
*Linanthus parryae* is a minute annual wildflower, widespread in the Mojave Desert. (A) Dissected *L. parryae* flowers, with the gynoecium, fused corolla and androecium, calyx, and subtending leaves separated and labeled. (B) Blue and white corolla polymorphic populations of *L. parryae* can be found throughout the species’ range. (C) A map of the species range including locations of herbarium specimens and observation reports aggregated by Calflora.org. The location of the specimen used for the genome assembly is marked in red. (D) A sandy wash west of Owens Lake, Inyo County, California, representative of *L. parryae* habitat.

The annual desert wildflower *Linanthus parryae* is an iconic species of the Mojave Desert. In years with higher rainfall, the species germinates prolifically and covers the desert floor with its well-known display of white and blue flowers. The species is endemic to California and occurs from 360 to 2100 m in elevation. Its geographic range extends across the western Mojave Desert, north through the Great Basin Desert of the Owens Valley, and in scattered populations across the Tehachapi Mountains and the southern inner CoastRange north to Shell Creek in San Luis Obispo County (see Fig. 1; Jepson Flora Project 2022). The species can be abundant in years of high rainfall, while in dry years the seeds remain dormant in the soil seedbank. *L. parryae* germinates in the winter and completes its life cycle by May.

One of the best-known attributes of this small desert plant is the color polymorphism that it displays both within populations and across its range. Populations are white flowered, blue-purple flowered, or polymorphic with both blue and white flowers often co-occurring in the same local habitat patch. Based on an unpublished herbarium survey, we found that approximately 40% of populations were polymorphic, and that these populations were distributed throughout the range of the species.

This extreme level of flower color polymorphism in *L. parryae* has attracted the attention of evolutionary biologists for generations and served as a model in debates about the degree to which natural selection or genetic drift contribute to the evolution of adaptively important characters in nature (Schemske and Bierzychudek 2007; Ishida 2017). In a highly influential paper, Epling and Dobzhansky mapped the relative frequencies of blue and white flowers across populations in the western Mojave Desert and concluded that populations with different color morph frequencies must represent subtly differentiated “microgeographic races” with relatively restricted gene flow between them (Epling and Dobzhansky 1942). These data were then used to test Wright’s shifting balance theory of evolution and assess the sometimes antagonistic roles of genetic drift and natural selection in maintaining intraspecific polymorphisms (Wright 1931, 1937). The *L. parryae* color polymorphism data were also used in the first empirical tests of models of isolation by distance (IBD) and were fundamental to the development of Wright’s *F* statistics that describe the genetic structure of natural populations to infer migration frequencies, inbreeding coefficients, and other aspects of demographic history (Wright 1943). Later studies proposed that natural selection linked to variation in rain regimes is the main evolutionary force maintaining *L. parryae* flower color polymorphism (Schemske and Bierzychudek 2001, 2007). Given the importance of this species in these foundational studies, modern genomic approaches seem to be the inevitable next step in examining the genetic basis and molecular mechanisms underlying this flower color polymorphism in this classic evolutionary genetic model system. These studies could also be expanded to include other flower color polymorphic species in the genus *Linanthus* including *L. dianthiflorus*, *L. killipii*, *L. bernardinus*, and *L. orcuttii*. Exploring the genetic basis for polymorphisms across closely related species and the roles of selection, drift, and gene flow operating on their respective landscapes will shed light on how polymorphisms arise and are maintained in nature.

Despite the central role of *L. parryae* in the development of phylogeography, landscape genetics, and population genetics, its habitat is in peril. Large swaths of the Mojave Desert are being developed for housing and photovoltaic power grids and utilized by Off-Highway Vehicles (OHVs). These flat sandy areas often coincide with the habitat preferred by desert annuals including *L. parryae*. Additionally, increases in temperature associated with global climate change can lead to range shifts and local extinctions for desert species that already occupy the upper extremes of plant thermal tolerance (Osmond *et al.* 1987; Lenoir and Svenning 2015). For such desert species to survive a changing climate, they must shift their range much faster than species in coastal or mountainous regions, which may be difficult to achieve in the shallow climatic gradients that characterize much of the desert (Loarie *et al.* 2009). At the same time, wetter microhabitats seem to be correlated with the ability of desert species to tolerate higher temperatures (Curtis *et al.* 2016). The large range of *L. parryae* throughout arid areas of California coupled with its adaptation to fluctuating patterns of rainfall make it an ideal species to study the ways that small desert annuals adapt to climate change, and to inform decision-makers on the extent to which protected areas will effectively support the survival of desert species. Understanding how desert annual species respond to global climatic changes and habitat destruction is therefore crucial to the conservation and protection of the desert biome that occupies 38% of California (Mooney and Zavaleta 2016).

This study reports the first chromosome-level genome assembly of *L. parryae*. To our knowledge, this is the second chromosome-level genome in the family Polemoniaceae (Jarvis *et al.* 2022), a group of significant historical importance in plant biosystematics and evolutionary biology (Grant and Grant 1965). This project was completed as part of the California Conservation Genomics Project (CCGP), an initiative with the goal of assembling genomic resources of endemic species in the state to inform conservation and management efforts (Fiedler *et al.* 2022; Shaffer *et al.* 2022). This reference genome will provide crucial resources to study the genetic mechanisms underlying and maintaining flower color polymorphism in the species. It will also serve as the foundation for studies identifying hotspots of genetic diversity and connectivity, assessing the genomic health of *L. parryae*, and investigating the genomic basis of adaptation to extreme environments.

## Methods

### Biological materials

We collected *L. parryae* individuals from a population with only white-flowered morphs. The 2 individuals sequenced in this project were collected on 17 May 2020, in the town of Phelan in the Mojave Desert, San Bernardino County, California. The location of the population was roadside, north of Muscatel Street, west of the intersection with Windemere Road, near the powerline, approximately 1 mile south of Phelan Road (34.412122° N, −117.493026° W). We collected entire plants, including leaves, flowers, stems, and roots. The plants were in the budding and flowering developmental stage. Immediately after collection, we placed each individual in separate 15 mL Nalgene bottles and kept the bottles in a liquid nitrogen dewar while in the field. The 2 individual plants were then stored in a −80 °C freezer. Individual plant IGA184.5 was shipped to the University of California Davis for high molecular weight (HMW) DNA extractions and Pacific BioSciences HiFi library preparation and sequencing (Pacific BioSciences—PacBio, Menlo Park, CA). Individual plant IGA184.4 was sent to University of California Santa Cruz for Omni-C library preparation and sequencing. A voucher for this population was previously collected (accession number UCR-112367) and it is stored at the herbarium of University of California, Riverside (UCR).

### Nucleic acid library preparation and sequencing

#### Omni-C library preparation and sequencing

The Omni-C library was prepared using the Dovetail Omni-C Kit (Dovetail Genomics, Scotts Valley, CA) according to the manufacturer’s protocol with slight modifications. First, specimen tissue from the whole plant (UCR112367; IGA184.4) was thoroughly ground with a mortar and pestle while cooled with liquid nitrogen. Nuclear isolation was then performed using published methods (Inglis *et al.* 2018). Subsequently, chromatin was fixed in place in the nucleus and digested under various conditions of DNase I until a suitable fragment length distribution of DNA molecules was obtained. Chromatin ends were repaired and ligated to a biotinylated bridge adapter followed by proximity ligation of adapter-containing ends. After proximity ligation, crosslinks were reversed, and the DNA purified from proteins. Purified DNA was treated to remove biotin that was not internal to ligated fragments. An NGS library was generated using an NEB Ultra II DNA Library Prep kit (New England Biolabs, Ipswich, MA) with an Illumina-compatible y-adaptor, biotin-containing fragments were captured using streptavidin beads, and the postcapture product was split into 2 replicates prior to PCR enrichment to preserve library complexity, with each replicate receiving unique dual indices. The library was sequenced at Vincent J. Coates Genomics Sequencing Lab (Berkeley, CA) on an Illumina NovaSeq (Illumina, CA) platform to generate approximately 100 million 2 × 150 bp read pairs per Gb genome size.

#### DNA extraction

We extracted HMW genomic DNA (gDNA) from whole plant tissue of a single individual (600 mg; IGA184.5) using the method described in Inglis *et al.* (2018), with the following modifications. We used sodium metabisulfite (1%, w/v) instead of 2-mercaptoethanol (1%, v/v) in the sorbitol wash buffer and the lysis buffer. Using mortar and pestle, we pulverized the frozen tissue in liquid nitrogen for 15 min, then gently resuspended it in 10 mL of sorbitol wash buffer. The suspension was centrifuged at 3000 × *g* for 5 min at room temperature, and the supernatant was discarded. Using a paintbrush, we gently resuspended the ground tissue pellet in 10 mL of sorbitol wash buffer and repeated the wash step 5 times to remove potential contaminants that may coprecipitate with DNA. We performed the lysis step at 45 °C (instead of the standard 65 °C, to avoid potential heat-induced DNA damage) for 1 h with gentle inversion every 15 min. The DNA purity was estimated using absorbance ratios (260/280 = 1.80 and 260/230 = 2.24) on a NanoDrop ND-1000 spectrophotometer. The final DNA yield (84 ng/µL; 32 µg) was quantified using a Quantus Fluorometer (QuantiFluor ONE dsDNA Dye assay; Promega, Madison, WI). The size distribution of the HMW DNA was estimated using the Femto Pulse system (Agilent, Santa Clara, CA), where 52% of the DNA fragments were found to be 45 kb or longer.

#### HiFi library preparation and sequencing

The HiFi SMRTbell library was constructed using the SMRTbell Express Template Prep Kit v2.0 (PacBio, Cat. #100-938-900) according to the manufacturer’s instructions. HMW gDNA was sheared to a target DNA size distribution between 15 and 20 kb. The sheared gDNA was concentrated using 0.45 of AMPure PB beads (PacBio, Cat. #100-265-900) for the removal of single-strand overhangs at 37 °C for 15 min, followed by further enzymatic steps of DNA damage repair at 37 °C for 30 min, end repair and A-tailing at 20 °C for 10 min and 65 °C for 30 min, ligation of overhang adapter v3 at 20 °C for 60 min and 65 °C for 10 min to inactivate the ligase, then nuclease treated at 37 °C for 1 h. The SMRTbell library was purified and concentrated with 0.45 Ampure PB beads (PacBio, Cat. #100-265-900) for size selection using the BluePippin/PippinHT system (Sage Science, Beverly, MA; Cat #BLF7510/HPE7510) to collect fragments greater than 7 to 9 kb. The 15 to 20 kb average HiFi SMRTbell library was sequenced at UC Davis DNA Technologies Core (Davis, CA) using 3 8M SMRT cells, Sequel II sequencing chemistry 2.0, and 30-h movies each on a PacBio Sequel II sequencer.

### Nuclear genome assembly

We assembled the *L. parryae* genome following the CCGP assembly protocol Version 4.0, as outlined in Table 1 which lists the nondefault parameters used in the assembly. As with other CCGP assemblies, our goal was to produce a high-quality and highly contiguous assembly using PacBio HiFi reads and Omni-C data while minimizing manual curation. We removed remnant adapter sequences from the PacBio HiFi dataset using HiFiAdapterFilt (Sim *et al.* 2022) and obtained the dual or partially phased initial diploid assembly (http://lh3.github.io/2021/10/10/introducing-dual-assembly) using HiFiasm (Cheng *et al.* 2021). We tagged output haplotype 1 as the primary assembly, and output haplotype 2 as the alternate assembly. We aligned the Omni-C data to the assemblies by using the Arima Genomics Mapping Pipeline (https://github.com/ArimaGenomics/mapping_pipeline) and then scaffolded both assemblies with SALSA (Ghurye *et al.* 2017, 2018). Next, we identified sequences corresponding to haplotypic duplications, contig overlaps and repeats on the primary assembly with purge_dups (Guan *et al.* 2020) and transferred them to the alternate assembly.

**Table 1.** Assembly pipeline and software used.

Assembly	Software	Version
Filtering PacBio HiFi adapters	HiFiAdapterFilt	Commit 64d1c7b
K-mer counting	Meryl (k = 21)	1
Estimation of genome size and heterozygosity	GenomeScope	2
De novo assembly (contigging)	HiFiasm (Hi-C mode, –primary, output p_ctg.hap1, p_ctg.hap2)	0.16.1-r375
Remove low-coverage, duplicated contigs	purge_dups	1.2.6
Scaffolding		
Omni-C data alignment for SALSA	Arima Genomics Mapping Pipeline (https://github.com/ArimaGenomics/mapping_pipeline)	Commit 2e74ea4
Omni-C scaffolding	SALSA (-DNASE, -i 20, -p yes)	2
Gap closing	YAGCloser (-mins 2 -f 20 -mcc 2 -prt 0.25 -eft 0.2 -pld 0.2)	Commit 20e2769
OmniC contact map generation		
Short-read alignment	BWA-MEM (-5SP)	0.7.17-r1188
SAM/BAM processing	Samtools	1.11
SAM/BAM filtering	pairtools	0.3.0
Pairs indexing	pairix	0.3.7
Matrix generation	Cooler	0.8.10
Matrix balancing	HiCExplorer (hicCorrectmatrix correct --filterThreshold -2 4)	3.6
Contact map visualization	HiGlass	2.1.11
	PretextMap	0.1.4
	PretextView	0.1.5
	PretextSnapshot	0.03
Benchmarking		
Basic assembly stats	QUAST (--est-ref-size)	5.0.2
Assembly completeness	BUSCO (-m geno, -l embryophyta)	5.0.0
	Merqury	1
Contamination screening		
General contamination screening	BlobToolKit	2.3.3
Local alignment tool	BLAST+	2.10
Repeat element identification		
Repeat identification	RepeatModeler	2.0.3
Repeat annotation	RepeatMasker	4-1-2

Software citations are listed in the text.

We generated Omni-C contact maps for both assemblies by aligning the Omni-C data against the corresponding assembly with BWA-MEM (Li 2013), identified ligation junctions, and generated Omni-C pairs using pairtools (Goloborodko*et al.* 2018). We generated a multiresolution Omni-C matrix with cooler (Abdennur and Mirny 2020) and balanced it with hicExplorer (Ramírez *et al.* 2018). We used HiGlass (Kerpedjiev *et al.* 2018) and the PretextSuite (https://github.com/wtsi-hpag/PretextView; https://github.com/wtsi-hpag/PretextMap; https://github.com/wtsi-hpag/PretextSnapshot) to visualize the contact maps, then checked the contact maps for major misassemblies. In detail, if in the proximity of a join that was made by the scaffolder, we identified a strong signal off-diagonal and lack of signal in the consecutive genomic region, we marked this join. All the joins that were marked, were “dissolved”, meaning that we broke the scaffolds at the coordinates of these joins. After this process, no further joins were made. Using the PacBio HiFi reads and YAGCloser (https://github.com/merlyescalona/yagcloser), we closed some of the remaining gaps generated during scaffolding. We then checked for contamination using the BlobToolKit Framework (Challis *et al.* 2020). Finally, we trimmed remnants of sequence adaptors and mitochondrial contamination identified during the contamination screening performed by NCBI.

### Genome size estimation and quality assessment

We generated k-mer counts from the PacBio HiFi reads using meryl (https://github.com/marbl/meryl). The k-mer database was then used in GenomeScope 2.0 (Ranallo-Benavidez*et al.* 2020) to estimate genome features including genome size, heterozygosity, and repeat content. To obtain general contiguity metrics, we ran QUAST (Gurevich *et al.* 2013). To evaluate genome quality and completeness we used BUSCO (Manni *et al.* 2021) with the embryophyta ortholog database (embryophyta_odb10) which contains 1,614 genes. Assessment of base level accuracy (QV) and k-mer completeness was performed using the previously generated meryl database and merqury (Rhie *et al.* 2020). We further estimated genome assembly accuracy via BUSCO gene set frameshift analysis using the pipeline described in Korlach *et al.* (2017). We identified and annotated repeat sequences using RepeatModeler and RepeatMasker (Smit and Hubley 2008–2015; Smit *et al.* 2013–2015).

Measurements of the size of the phased blocks are based on the size of the contigs generated by HiFiasm in HiC mode. We followed the quality metric nomenclature established by Rhie *et al.* (2021), with the genome quality code *x*·*y*·*P*·*Q*·*C*, where *x* = log10[contig NG50]; *y* = log10[scaffold NG50]; *P* = log10[phased block NG50]; *Q* = Phred base accuracy QV (quality value); *C* = % genome represented by the first “*n*” scaffolds, following a known karyotype 2*n* = 18 (Patterson 1979). Quality metrics for the notation were calculated on the primary assembly.

## Results

**Fig. 2.**
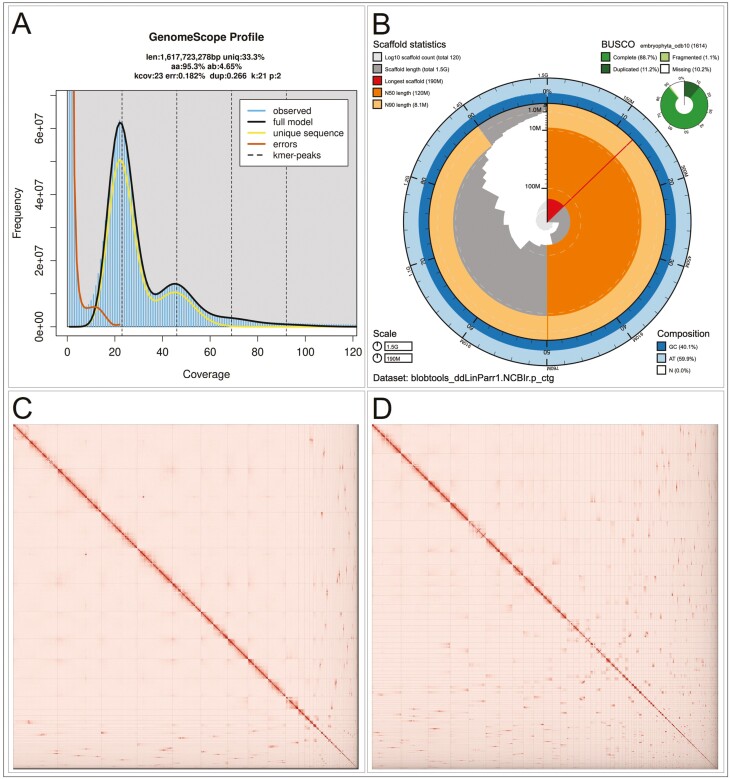
Visual overview of the *Linanthus parryae* genome assembly metrics. (A) K-mer spectra output generated from PacBio HiFi data without adapters using GenomeScope 2.0. The bimodal pattern observed corresponds to a diploid genome and the k-mer profile matches that of high heterozygosity. K-mers at lower coverage and high frequency correspond to differences between haplotypes, whereas the higher coverage and low frequency k-mers correspond to the similarities between haplotypes. (B) BlobToolKit snail plot showing a graphical representation of the quality metrics presented in Table 2 for the *Linanthus parryae* primary assembly (ddLinParr1.0.p). The plot circle represents the full size of the assembly. From the inside-out, the central plot covers length-related metrics. The red line represents the size of the longest scaffold; all other scaffolds are arranged in size-order moving clockwise around the plot and drawn in gray starting from the outside of the central plot. Dark and light orange arcs show the scaffold N50 and scaffold N90 values. The central light gray spiral shows the cumulative scaffold count with a white line at each order of magnitude. White regions in this area reflect the proportion of Ns in the assembly; the dark versus light blue area around it shows mean, maximum and minimum GC versus AT content at 0.1% intervals (Challis *et al.* 2020). Hi-C contact maps for the primary (C) and alternate (D) genome assembly generated with PretextSnapshot. Hi-C contact maps translate proximity of genomic regions in 3D space to contiguous linear organization. Each cell in the contact map corresponds to sequencing data supporting the linkage (or join) between 2 of such regions. Scaffolds are separated by black lines and higher density of the lines may correspond to higher levels of fragmentation.

The Omni-C and PacBio HiFi sequencing libraries generated 295.4 million read pairs and 4.8 million reads, respectively. The latter yielded 39.39-fold coverage (N50 read length 16,891 bp; minimum read length 48 bp; mean read length 16,034 bp; maximum read length 58,036 bp). Calculation of coverage is based on a flow cytometry estimated genome size of 1.96 Gb. The GenomeScope 2.0 genome size estimation was 1.6 Gb. Based on PacBio HiFi reads, we estimated a 0.182% sequencing error rate and 4.65% nucleotide heterozygosity rate. The k-mer spectrum based on PacBio HiFi reads (Fig. 2A) shows a bimodal distribution with 2 major peaks at ~23- and ~45-fold coverage, where peaks correspond to homozygous and heterozygous states of a diploid species, respectively. The distribution presented in this k-mer spectrum supports that of a high heterozygosity profile.

The final assembly (ddLinParr1) consists of 2 pseudo haplotypes, primary and alternate, both genome sizes similar to the estimated value from GenomeScope 2.0 (Fig. 2A). The primary assembly consists of 123 scaffolds spanning 1.5 Gb with a contig N50 of 18.7 Mb, scaffold N50 of 124.8 Mb, largest contig of 69.4 Mb and largest scaffold of 194.5 Mb. The alternate assembly consists of 581 scaffolds, spanning 1.8 Gb with contig N50 of 10.6 Mb, scaffold N50 of 44.0 Mb, largest contig 41.8 Mb and largest scaffold of 145.2 Mb. Detailed assembly statistics are reported in Table 2, and a graphical representation for the primary assembly in Fig. 2B (see Supplementary Fig. 1 for the alternate assembly). The Omni-C contact map suggests that the primary assembly is highly contiguous (Fig. 2C).

We identified a total of 12 misassemblies, 6 per assembly, and broke the corresponding joins made by SALSA2 on both assemblies. We were able to close a total of 22 gaps, 9 on the primary and 13 on the alternate assembly. Finally, we filtered 15 contigs from the primary assembly, 1 matching to Oomycota and the rest to Ascomycota contaminants. No further contigs were removed. The primary assembly has a BUSCO completeness score of 88.7% using the embryophyta gene set, a per base quality (QV) of 68.93, a k-mer completeness of 58.96 and a frameshift indel QV of 46.04. The alternate assembly has a BUSCO completeness score of 84.4% using the embryophyta gene set, a per base quality (QV) of 66.36, a k-mer completeness of 60.95 and a frameshift indel QV of 47.1. We have deposited scaffolds corresponding to both primary and alternate assemblies on NCBI (see Table 2 and Data availability for details).

**Table 2.** Sequencing and assembly statistics, and accession numbers.

BioProjects and vouchers	CCGP NCBI BioProject			PRJNA720569			
	Genera NCBI BioProject			PRJNA765619			
	Species NCBI BioProject			PRJNA777190			
	NCBI BioSample			SAMN26264369			
	Specimen identification			IGA184			
	NCBI Genome accessions			Primary		Alternate	
	Assembly accession			JALGPY000000000		JALGPZ000000000	
	Genome sequences			GCA_023055425.1		GCA_023055565.1	
Genome sequence	PacBio HiFi reads	Run		1 PACBIO_SMRT (Sequel II) run: 4.8M spots, 77.2G bases, 52.5 Gb			
		Accession		SRX15304035			
	Omni-C Illumina reads	Run		1 ILLUMINA (Illumina NovaSeq 6000) run: 295.4M spots, 65.3G bases, 21.4G			
		Accession		SRX15304036, SRX15304037			
Genome Assembly Quality Metrics	Assembly identifier (quality code^a^)			ddLinParr1 (7.8.P7.Q67.C78)			
	HiFi read coverage^b^			39.39×			
				Primary		Alternate	
	Number of contigs			208		730	
	Contig N50 (bp)			18,687,320		10,572,045	
	Contig NG50			17,395,258		11,393,586	
	Longest contigs			69,385,222		41,847,382	
	Number of scaffolds			123		581	
	Scaffold N50			124,808,130		43,960,851	
	Scaffold NG50			124,808,130		47,495,494	
	Largest scaffold			194,533,388		145,210,036	
	Size of final assembly (bp)			1,514,484,308		1,755,711,046	
	Phase block NG50			17,395,258		11,393,586	
	Gaps per Gbp (#Gaps)			56 (85)		85 (149)	
	Indel QV (frameshift)			46.04081985		47.09876692	
	Base pair QV			68.9289		66.3567	
						Full assembly = 67.3638	
	K-mer completeness			58.9657		60.9501	
						Full assembly = 98.0448	
	BUSCO completeness (embryophyta), *n* = 1,614		C	S	D	F	M
		P^c^	88.70%	77.50%	11.20%	1.10%	10.20%
		A^c^	84.40%	74.50%	9.90%	1.10%	14.50%

Assembly quality code *x*·*y*·*P*·*Q*·*C*, where *x* = log10[contig NG50]; *y* = log10[scaffold NG50]; *P* = log10[phased block NG50]; *Q* = Phred base accuracy QV (quality value); *C* = % genome represented by the first “*n*” scaffolds, following a known karyotype 2*n* = 18 (Patterson 1979). BUSCO scores. (C)omplete and (S)ingle; (C)omplete and (D)uplicated; (F)ragmented and (M)issing BUSCO genes. *n*, number of BUSCO genes in the set/database. Bp: base pairs.

Read coverage and NGx statistics have been calculated based on a genome size of 1.96 Gb.

P(rimary) and (A)lternate assembly values.

## Discussion

Prior to sequencing, we estimated the genome size of *L. parryae* using flow cytometry. Our 1C estimates were between 1.93 and 1.99 Gb. This difference in size compared with the GenomeScope estimation of 1.6 Gb may be due to repetitive elements in the genome. Based on flow cytometry, the species seems to have an average genome size compared with other species in the genus. Flow cytometry genome size estimations of 9 other species of *Linanthus* ranged from 1.31 to 3.51 Gb, with an average of 2.02 Gb.

This assembly is the second published genome in the family Polemoniaceae, and the first for *Linanthus*. The other chromosome-level genome assembly in the family is *Gilia yorkii*. *Gilia* and *Linanthus* last shared a common ancestor about 60 MYA (Landis *et al.* 2018). While these species have not shared an evolutionary history for a long period of time, we provide these metrics as a context for comparative genomics in Polemoniaceae. *G. yorkii* was sequenced using PacBio at 67× coverage compared with the *L. parryae* at 39.4× coverage. The genome size of *G. yorkii* was estimated at 2.80 Gb and the *L. parryae* at 1.96 Gb. The BUSCO completeness score for *G. yorkii* was 96.8% compared with the 88.7% in *L. parryae*. The *L. parryae* assembly has a longer contig N50 than the *G. yorkii* genome (18.7 vs. 2.5 Mb) and a shorter scaffold N50 (76.8 vs. 285.8 Mb). The *G. yorkii* reads were assembled in 3,947 contigs and 2,043 scaffolds, compared with 208 contigs and 123 scaffolds for *L. parryae* (Jarvis *et al.* 2022).

A total of 71.99% of the *L. parryae* genome was annotated as repetitive. This is similar to the values reported for *G. yorkii* (75.60%), but higher than the average of 45.49% across plant species (Luo *et al.* 2022). Long-terminal repeat (LTR) retroelements made up 38.26% of the genome, less than for *G. yorkii* at 45.81% and more than the plant species average at 21.66%. Of the LTRs, Ty1/Copia made up 24.3% of the genome, which is similar to *G. yorkii* at 29.43%. Over a quarter of the genome (26.62%) was annotated as unclassified repeat elements, which is again similar to *G. yorkii* (21.92%); both species are about double the plant average (13.2%). This high percentage of unknown repeats may reflect the fact that plants in general, and Polemoniaceae in particular, are not well represented in the repeat database (Luo *et al.* 2022). The RepeatMasker annotation of repeat elements for *L. parryae* is summarized in Table 3.

**Table 3.** Classification of repeat elements generated from RepeatMasker.

	Number of elements^a^	Length occupied (bp)	Percentage of sequence (%)
Retroelements	649,089	1,119,332,631	42.05
SINEs	0	0	0.00
Penelope	0	0	0.00
LINEs	122,415	101,011,181	3.80
CRE/SLACS	0	0	0.00
L2/CR1/Rex	0	0	0.00
R1/LOA/Jockey	0	0	0.00
R2/R4/NeSL	0	0	0.00
RTE/Bov-B	7,307	1,975,180	0.07
L1/CIN4	115,108	99,036,001	3.72
LTR elements	526,674	1,018,321,450	38.26
BEL/Pao	0	0	0.00
Ty1/Copia	330,448	646,651,635	24.30
Gypsy/DIRS1	196,097	371,644,595	13.96
Retroviral	129	25,220	0.00
DNA transposons	62,196	51,067,856	1.92
hobo-Activator	15,958	5,783,269	0.22
Tc1-IS630-Pogo	3,961	1,515,451	0.06
En-Spm	0	0	0.00
MuDR-IS905	0	0	0.00
PiggyBac	0	0	0.00
Tourist/Harbinger	4,485	1,597,874	0.06
Other (Mirage, P-element, Transib)	0	0	0.00
Rolling-circles	4,483	5,689,821	0.21
Unclassified	1,755,132	708,389,341	26.62
Total interspersed repeats		1,878,789,828	70.59
Small RNA	22,615	11,470,955	0.43
Satellites	12,336	1,179,847	0.04
Simple repeats	357,637	16,506,411	0.62
Low complexity	48,902	2,518,203	0.09

Most repeats fragmented by insertions or deletions have been counted as one element.

This reference genome will contribute to conservation in California in several important ways. *L. parryae* occurs in a region of California considered a hotbed of plant diversity and neoendemism (Kraft *et al.* 2010). In concert with other species in the CCGP, this genomic resource will help determine whether these geographic areas also correspond to hotspots of genomic diversity and provide important information for setting realistic priorities for conservation. Although a desert species, *L. parryae* occurs across a relatively wide and heterogeneous environment gradient with an elevational range of 360 to 2100 m, a precipitation range of 50 to 500 mm/yr, and a geographic range that spans 470 km. The morphological and environmental variation along this species distribution may correspond with genetic differentiation between populations and potential local adaptation. Hence, this genome can serve as a foundation to investigate patterns of genomic diversity, connectivity, and health for other California plants, as well as shed light on the sorting of adaptive genomic variation across ecological gradients that are often associated with species divergence.

*L. parryae* occurs in fragile habitats that are particularly prone to habitat destruction, with at least 5 major ongoing threats. First, large swaths of the desert are now utilized by OHVs, including 200,000 acres of newly designated motorized recreation land established by the California Desert Protection Act of 2019 (Feinstein 2020). Second, photovoltaic power grid development is increasing with projects proposed or being built on an additional 30,000 acres of California desert (Wilson 2020). These developments can, and will, drastically impact unprotected desert lands (Hernandez *et al.* 2015). Third, urban sprawl spilling over from the Los Angeles Basin has increased habitat destruction in the southwestern edge of the Mojave Desert (Stewart 1997), an area with the highest density of *L. parryae*. Fourth, as water resources become more scarce and seasonal streams are diverted, ephemeral wash habitat for *Linanthus* species, as well as other desert dwellers, may become uninhabitable (Levick *et al.* 2008). Lastly, ecological pressure from ongoing invasive plants will be exacerbated with climate change (Smith *et al.* 2000). This reference genome will help determine the effects of these threats and enhance conservation plans in currently protected desert areas, helping resource managers determine which natural areas to prioritize for future protection.

## Supplementary Material

esac052_suppl_Supplementary_Figure_Legend

esac052_suppl_Supplementary_Figure_S1

## Data Availability

Data generated for this study are available under NCBI BioProject PRJNA777190. Raw sequencing data for sample IGA184 (NCBI BioSample SAMN26264369) are deposited in the NCBI Short Read Archive (SRA) under SRX15304035 for PacBio HiFi sequencing data, and SRX15304036, SRX15304037 for the Omni-C Illumina sequencing data. GenBank accessions for both primary and alternate assemblies are GCA_023055425.1 and GCA_023055565.1; and for genome sequences JALGPY000000000 and JALGPZ000000000. Assembly scripts and other data for the analyses presented can be found at the following GitHub repository: www.github.com/ccgproject/ccgp_assembly.

## References

[CIT0001] Abdennur N , Mirny LA. Cooler: scalable storage for Hi-C data and other genomically labeled arrays. Bioinformatics. 2020;36(1):311–316. 31290943 10.1093/bioinformatics/btz540 PMC8205516

[CIT0002] Challis R , Richards E, Rajan J, Cochrane G, Blaxter M. BlobToolKit—interactive quality assessment of genome assemblies. G3. 2020;10(4):1361–1374. 32071071 10.1534/g3.119.400908 PMC7144090

[CIT0003] Cheng H , Jarvis ED, Fedrigo O, Koepfli K-P, Urban L, Gemmell NJ, Li H. Robust haplotype-resolved assembly of diploid individuals without parental data, arXiv [q-bio.GN], arXiv:2109.04785, 2021, preprint: not peer reviewed. doi:10.48550/arXiv.2109.04785 PMC9464699 35332338

[CIT0004] Curtis EM , Gollan J, Murray BR, Leigh A. Native microhabitats better predict tolerance to warming than latitudinal macro-climatic variables in arid-zone plants. J Biogeogr. 2016;43(6):1156–1165.

[CIT0005] Epling C , Dobzhansky T. Genetics of natural populations. VI. Microgeographic races of *Linanthus parryae*. Genetics. 1942;27(3):317–332. 17247043 10.1093/genetics/27.3.317 PMC1209161

[CIT0006] Feinstein D. United States Senator for California. 2020 [accessed 2020 Mar 9]. https://www.feinstein.senate.gov/public/index.cfm/2019/2/senate-passes-feinstein-bill-completing-25-year-effort-to-protect-california-desert

[CIT0007] Fiedler PL , Erickson B, Esgro M, Gold M, Hull JM, Norris J, Shapiro B, Westphal MF, Toffelmier E, Shaffer HB. Seizing the moment: the opportunity and relevance of the California Conservation Genomics Project to state and federal conservation policy. J Hered. 2022; esac046. doi:10.1093/jhered/esac046 36136001 PMC9709969

[CIT0008] Ghurye J , Pop M, Koren S, Bickhart D, Chin C-S. Scaffolding of long read assemblies using long range contact information. BMC Genomics. 2017;18(1):1–11. 28701198 10.1186/s12864-017-3879-z PMC5508778

[CIT0009] Ghurye J , Rhie A, Walenz BP, Schmitt A, Selvaraj S, Pop M, Koren S. Integrating Hi-C links with assembly graphs for chromosome-scale assembly. PLoS Comput Biol. 2018;15(8):e1007273. 10.1371/journal.pcbi.1007273 PMC6719893 31433799

[CIT0010] Goloborodko A , Abdennur N, Venev S, Hbbrandao, gfudenberg. mirnylab/pairtools: v0.2.0. 2018. https://zenodo.org/record/1490831

[CIT0011] Grant V , Grant KA. Flower pollination in the Phlox family. New York (NY): Columbia University Press; 1965.

[CIT0012] Guan D , McCarthy SA, Wood J, Howe K, Wang Y, Durbin R. Identifying and removing haplotypic duplication in primary genome assemblies. Bioinformatics. 2020;36(9):2896–2898. 31971576 10.1093/bioinformatics/btaa025 PMC7203741

[CIT0013] Gurevich A , Saveliev V, Vyahhi N, Tesler G. QUAST: quality assessment tool for genome assemblies. Bioinformatics. 2013;29(8):1072–1075. 23422339 10.1093/bioinformatics/btt086 PMC3624806

[CIT0014] Hernandez RR , Hoffacker MK, Murphy-Mariscal ML, Wu GC, Allen MF. Solar energy development impacts on land cover change and protected areas. Proc Natl Acad Sci USA. 2015;112(44):13579–13584. 26483467 10.1073/pnas.1517656112 PMC4640750

[CIT0015] Inglis PW , Pappas MDCR, Resende LV, Grattapaglia D. Fast and inexpensive protocols for consistent extraction of high quality DNA and RNA from challenging plant and fungal samples for high-throughput SNP genotyping and sequencing applications. PLoS One. 2018;13(10):e0206085. 30335843 10.1371/journal.pone.0206085 PMC6193717

[CIT0016] Ishida Y. Sewall Wright, shifting balance theory, and the hardening of the modern synthesis. Stud Hist Philos Biol Biomed Sci. 2017;61:1–10. 27907853 10.1016/j.shpsc.2016.11.001

[CIT0017] Jarvis DE , Maughan PJ, DeTemple J, Mosquera V, Li Z, Barker MS, Johnson LA, Whipple CJ. Chromosome-scale genome assembly of *Gilia yorkii* enables genetic mapping of floral traits in an interspecies cross. Genome Biol Evol. 2022;14(3):1–13, evac017. 10.1093/gbe/evac017 PMC8920513 35106544

[CIT0018] Jepson Flora Project, editor. Jepson eFlora. 2022. Accessed on June 7, 2022. https://ucjeps.berkeley.edu/eflora/

[CIT0019] Kerpedjiev P , Abdennur N, Lekschas F, McCallum C, Dinkla K, Strobelt H, Luber JM, Ouellette SB, Azhir A, Kumar N, et al. HiGlass: web-based visual exploration and analysis of genome interaction maps. Genome Biol. 2018;19(1):1–12. 30143029 10.1186/s13059-018-1486-1 PMC6109259

[CIT0020] Korlach J , Gedman G, Kingan SB, Chin C-S, Howard JT, Audet J-N, Jarvis ED. De novo PacBio long-read and phased avian genome assemblies correct and add to reference genes generated with intermediate and short reads. GigaScience. 2017;6(10):1–16. 10.1093/gigascience/gix085 PMC5632298 29020750

[CIT0021] Kraft NJ , Baldwin BG, Ackerly DD. Range size, taxon age and hotspots of neoendemism in the California flora. Divers Distrib. 2010;16(3):403–413.

[CIT0022] Landis JB , Bell CD, Hernandez M, Zenil-Ferguson R, McCarthy EW, Soltis DE, Soltis PS. Evolution of floral traits and impact of reproductive mode on diversification in the phlox family (Polemoniaceae). Mol Phylogenet Evol. 2018;127:878–890. 29958983 10.1016/j.ympev.2018.06.035

[CIT0023] Lenoir J , Svenning JC. Climate-related range shifts—a global multidimensional synthesis and new research directions. Ecography. 2015;38(1):15–28.

[CIT0024] Levick LR , Goodrich DC, Hernandez M, Fonseca J, Semmens DJ, Stromberg JC, Tluczek M, Leidy RA, Scianni M, Guertin DP, et al. The ecological and hydrological significance of ephemeral and intermittent streams in the arid and semi-arid American Southwest. Washington, DC: US Environmental Protection Agency, Office of Research and Development; 2008.

[CIT0025] Li H. Aligning sequence reads, clone sequences and assembly contigs with BWA-MEM, arXiv, arXiv:1303.3997, 2013, preprint: not peer reviewed. doi:10.48550/arXiv.1303.3997

[CIT0026] Loarie SR , Duffy PB, Hamilton H, Asner GP, Field CB, Ackerly DD. The velocity of climate change. Nature. 2009;462(7276):1052–1055. 20033047 10.1038/nature08649

[CIT0027] Luo X , Chen S, Zhang Y. PlantRep: a database of plant repetitive elements. Plant Cell Rep. 2022;41(4):1163–1166. 34977976 10.1007/s00299-021-02817-y PMC9035001

[CIT0028] Manni M , Berkeley MR, Seppey M, Simão FA, Zdobnov EM. BUSCO update: novel and streamlined workflows along with broader and deeper phylogenetic coverage for scoring of eukaryotic, prokaryotic, and viral genomes. Mol Biol Evol. 2021;38(10):4647–4654. 34320186 10.1093/molbev/msab199 PMC8476166

[CIT0029] Mooney H , Zavaleta E. Ecosystems of California. Oakland, California: University of California Press; 2016.

[CIT0030] Osmond CB , Austin MP, Berry JA, Billings WD, Boyer JS, Dacey JWH, Winner WE. Stress physiology and the distribution of plants. BioScience.1987;37(1):38–48.

[CIT0031] Patterson R. Chromosome numbers in annual *Linanthus* species. Madrono. 1979:96–100.

[CIT0032] Ranallo-Benavidez TR , Jaron KS, Schatz MC. GenomeScope 2.0 and Smudgeplot for reference-free profiling of polyploid genomes. Nat Commun. 2020;11(1):1–10. 32188846 10.1038/s41467-020-14998-3 PMC7080791

[CIT0033] Ramírez F , Bhardwaj V, Arrigoni L, Lam KC, Grüning BA, Villaveces J, Habermann B, Akhtar A, Manke, T. 2018. High-resolution TADs reveal DNA sequences underlying genome organization in fies. Nat Commun. 9:1–5. 29335486 10.1038/s41467-017-02525-w PMC5768762

[CIT0034] Rhie A , McCarthy SA, Fedrigo O, Damas J, Formenti G, Koren S, Uliano-Silva M, Chow W, Fungtammasan A, Kim J, et al. Towards complete and error-free genome assemblies of all vertebrate species. Nature. 2021;592:737–746. 33911273 10.1038/s41586-021-03451-0 PMC8081667

[CIT0035] Rhie A , Walenz BP, Koren S, Phillippy AM. Merqury: reference-free quality, completeness, and phasing assessment for genome assemblies. Genome Biol. 2020;21(1):1–27. 10.1186/s13059-020-02134-9 PMC7488777 32928274

[CIT0036] Schemske DW , Bierzychudek P. Perspective: evolution of flower color in the desert annual *Linanthus parryae*: Wright revisited. Evolution. 2001;55(7):1269–1282. 11525452 10.1111/j.0014-3820.2001.tb00650.x

[CIT0037] Schemske DW , Bierzychudek P. Spatial differentiation for flower color in the desert annual *Linanthus parryae*: was Wright right? Evolution. 2007;61(11):2528–2543. 17894812 10.1111/j.1558-5646.2007.00219.x

[CIT0038] Shaffer HB , Toffelmier E, Corbett-Detig RB, Escalona M, Erickson B, Fiedler P, Gold M, Harrigan RJ, Hodges S, Luckau TK, et al. Landscape genomics to enable conservation actions: the California Conservation Genomics Project. J Hered. 2022; 113:578-588. 10.1093/jhered/esac020 35395669

[CIT0039] Sim SB , Corpuz RL, Simmonds TJ, Geib SM. HiFiAdapterFilt, a memory efficient read processing pipeline, prevents occurrence of adapter sequence in PacBio HiFi reads and their negative impacts on genome assembly. BMC Genomics. 2022;23:1–7. 35193521 10.1186/s12864-022-08375-1 PMC8864876

[CIT0040] Smit AFA , Hubley R. RepeatModeler Open-1.0. 2008–2015. Accessed July 19, 2022. http://www.repeatmasker.org

[CIT0041] Smit AFA , Hubley R, Green P. RepeatMasker Open-4.0. 2013–2015. Accessed July 19, 2022. http://www.repeatmasker.org

[CIT0042] Smith SD , Huxman TE, Zitzer SF, Charlet TN, Housman DC, Coleman JS, Fenstermaker LK, Seemann JR, Nowak RS. Elevated CO_2_ increases productivity and invasive species success in an arid ecosystem. Nature. 2000;408(6808):79–82. 11081510 10.1038/35040544

[CIT0043] Stewart WC. Bioregional demographic trends and implications for biodiversity. Sacramento: Fire and Resource Assessment Program; 1997.

[CIT0044] Wilson J. Solar surges in the California desert. So why are environmentalists upset? Palm Springs Desert Sun. Jan 3, 2020. 2020 [accessed 2020 Mar 9]. https://www.desertsun.com/story/news/environment/2020/01/03/solar-surges-california-desert-environment-trump/2665799001/

[CIT0045] Wright S. Evolution in Mendelian populations. Genetics. 1931;16(2):97–159. 17246615 10.1093/genetics/16.2.97 PMC1201091

[CIT0046] Wright S. The distribution of gene frequencies in populations. Proc Natl Acad Sci USA. 1937;23(6):307–320. 16577780 10.1073/pnas.23.6.307 PMC1076930

[CIT0047] Wright S. Isolation by distance. Genetics. 1943;28(2):114–138. 17247074 10.1093/genetics/28.2.114 PMC1209196

