# Supplementary material for: Reference genome of the color polymorphic desert annual plant sandblossoms, Linanthus parryae
Source: J Hered. 2022 Sep 15;113(6):712–21. doi: 10.1093/jhered/esac052 (PMC9709995; doi:10.1093/jhered/esac052)
Supplement: esac052_suppl_Supplementary_Figure_Legend [file esac052_suppl_supplementary_figure_legend.docx]

**Supplementary Material**

**Figure 1.** BlobToolKit Snail plot showing a graphical representation of the quality metrics presented in Table 2 for the *Linanthus parryae* alternate assembly (ddLinParr1.0.a). The plot circle represents the full size of the assembly. From the inside-out, the central plot covers length-related metrics. The red line represents the size of the longest scaffold; all other scaffolds are arranged in size-order moving clockwise around the plot and drawn in gray starting from the outside of the central plot. Dark and light orange arcs show the scaffold N50 and scaffold N90 values. The central light gray spiral shows the cumulative scaffold count with a white line at each order of magnitude. White regions in this area reflect the proportion of Ns in the assembly; the dark vs. light blue area around it shows mean, maximum and minimum GC vs. AT content at 0.1% intervals (Challis et al. 2020)
