# Supplementary material for: Reference genome of the color polymorphic desert annual plant sandblossoms, Linanthus parryae
Source: J Hered. 2022 Sep 15;113(6):712–21. doi: 10.1093/jhered/esac052 (PMC9709995; doi:10.1093/jhered/esac052)
Supplement: esac052_suppl_Supplementary_Figure_S1 [file esac052_suppl_supplementary_figure_s1.pdf]

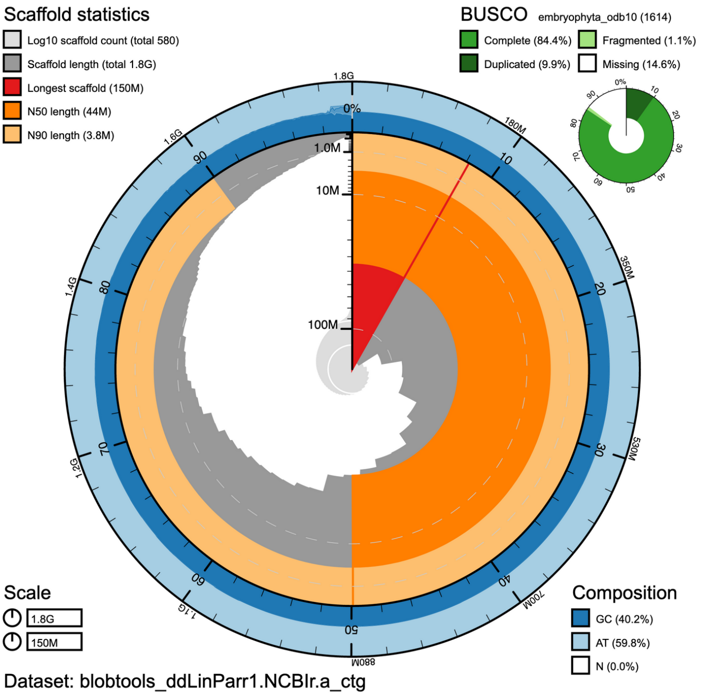

Scaffold statistics
Log10 scaffold count (total 580)
Scaffold length (total 1.8G)
Longest scaffold (150M)
N50 length (44M)
N90 length (3.8M)
BUSCO embryophyta_odb10 (1614)
Complete (84.4%)
Fragmented (1.1%)
Duplicated (9.9%)
Missing (14.6%)
Scale
1.8G
150M
Composition
GC (40.2%)
AT (59.8%)
N (0.0%)
Dataset: blobtools_ddLinParr1.NCBIr.a_ctg
